# High‐power, short‐duration ablation during Box isolation for atrial fibrillation

**DOI:** 10.1002/joa3.12407

**Published:** 2020-07-16

**Authors:** Koichiro Kumagai, Hideko Toyama

**Affiliations:** ^1^ Heart Rhythm Center Fukuoka Sanno Hospital International University of Health and Welfare Fukuoka Japan

**Keywords:** atrial fibrillation, Box isolation, catheter ablation, high‐power

## Abstract

**Background:**

It has been demonstrated that a high‐power, short‐duration (HPSD) ablation during pulmonary vein (PV) isolation is effective and safe. However, studies about the HPSD ablation during the posterior wall isolation, the Box isolation (BOXI), are limited. We evaluated the efficacy, feasibility, and safety of HPSD ablation during BOXI.

**Methods:**

One‐hundred sixty patients with all types of atrial fibrillation underwent BOXI with HPSD ablation (n = 80) or conventional technique (n = 80). In the HPSD group, ablation was performed with 50 W and a target lesion size index of 5.0 using a contact force (CF) sensing catheter. Ablation near the esophagus was performed with 50 W for 5 seconds and a CF < 10 g. In the conventional group, ablation was performed with 30‐40 W for 30 seconds, but 20 W near the esophagus.

**Results:**

The BOXI creation (26 ± 8 minutes vs 47 ± 17 minutes, *P* < .0001) and procedure (65 ± 12 minutes vs 87 ± 23 minutes, *P* < .0001) times were significantly shorter in the HPSD group than the conventional group. The number of pacing capture sites did not differ between the two groups. No complications including gastrointestinal symptoms occurred. The atrial tachyarrhythmia‐free rate at 12‐months after a single procedure was 86.3% in the HPSD group and 76.3% in the conventional group, respectively (*P* = .132). The incidence of PV reconnections and gaps in the lines during the second procedure did not differ between the two groups.

**Conclusion:**

The BOXI with HPSD ablation is effective, feasible, and safe with short BOXI creation and procedure times without reducing the clinical outcomes.

## Graphical abstract

The Box isolation (BOXI) with high‐power, short‐duration ablation is effective, feasible, and safe with short BOXI creation and procedure times without reducing the clinical outcomes.

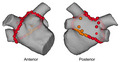

## 1 INTRODUCTION

Radiofrequency (RF) catheter ablation of atrial fibrillation (AF) has been widely performed.^1^ In persistent AF, the additional benefit of a posterior wall box isolation (BOXI) over the pulmonary vein isolation (PVI) has been reported in previous studies.^2^, ^3^, ^4^, ^5^, ^6^, ^7^, ^8^, ^9^, ^10^, ^11^, ^12^

Although a durable lesion formation of the PVI or BOXI is essential for successful outcomes, ablation on the posterior wall has a risk of esophageal injury. A lower‐power, long‐duration (LPLD) ablation (10‐35 W for 10‐30 seconds) is commonly used on the posterior wall.^13^, ^14^ In contrast, a high‐power, short‐duration (HPSD) ablation (50 W for 2‐15 seconds) during the PVI has been advocated to decrease the procedure time without an increase in complications.^15^, ^16^, ^17^, ^18^, ^19^ However, the optimal RF power and duration settings on the posterior wall near the esophagus are not fully elucidated and studies about HPSD ablation during BOXI are limited.

Therefore, we evaluated the efficacy, feasibility, and safety of HPSD ablation using contact force (CF) sensing catheters during BOXI as compared to that with the conventional technique.

## 2 METHODS

One‐hundred sixty consecutive patients with AF underwent catheter ablation from June 2018 to May 2019. The patients were divided into two groups: HPSD ablation group (n = 80); and conventional group (n = 80). We performed conventional methods in the first 80 patients, then HPSD methods in the last 80 patients. The clinical characteristics of each group are shown in Table 1.

**TABLE 1:** Patient characteristics

	HPSD (n = 80)	Conventional (n = 80)	*P*
Age, y	63.0 ± 9.1	63.1 ± 9.1	.958
Female, n (%)	20 (25)	14 (17.5)	.334
CHADS_2_ score	0.7 ± 1.0	0.8 ± 0.8	.798
AF type			.599
Paroxysmal AF, n (%)	30 (37.5)	24 (30.0)	
Persistent AF, n (%)	23 (28.8)	25 (31.3)	
Longstanding AF, n (%)	27 (33.8)	31 (38.8)	
LA diameter, mm	41.6 ± 5.1	43.3 ± 6.4	.069
LVEF, %	62.5 ± 7.7	62.2 ± 7.2	.832

Abbreviations: AF, atrial fibrillation; HPSD, high‐power short‐duration; LA, left atrium; LVEF, left ventricular ejection fraction.

The AF type was defined as paroxysmal lasting <1 week, persistent lasting >1 week, and <1 year or requiring pharmacologic or electrical cardioversion in <1 week, or longstanding persistent lasting >1 year. Written informed consent was obtained from all patients. The study was approved by the Fukuoka Sanno Hospital's Institutional Review Board.

All patients underwent the catheter ablation under deep sedation with dexmedetomidine, fentanyl, and thiopental. A 7F duodecapolar catheter (Bee‐AT; Japan‐Lifeline Co., Ltd.) was placed in the coronary sinus from the right internal jugular vein. The transseptal puncture was performed using an NRG RF needle (Baylis Medical). A 20‐pole circular mapping catheter (Optima^TM^; Abbott) and irrigated‐tip ablation catheter (FlexAbility^TM^ or TactiCath^TM^ CF sensing catheter; Abbott) were used for mapping and ablation. The EnSite NavX^TM^ system (Abbott) was used. Esophageal temperature monitoring was performed using a probe (SensiTherm ^TM^; Abbott).

### 2.1 BOXI with HPSD ablation

All ablation applications except for those near the esophagus were delivered with 50 W and a target lesion size index (LSI) of 5.0 using the TactiCath^TM^ CF sensing catheter with a target CF of 5‐20 g. Ablation applications near the esophagus were delivered with 50 W for 5 seconds with a CF of <10 g, but the application was stopped when the esophageal temperature increased to 40°C. We used a catheter irrigation rate of 30 mL/min and temperature cutoff of 42°C.

**FIGURE 1:**
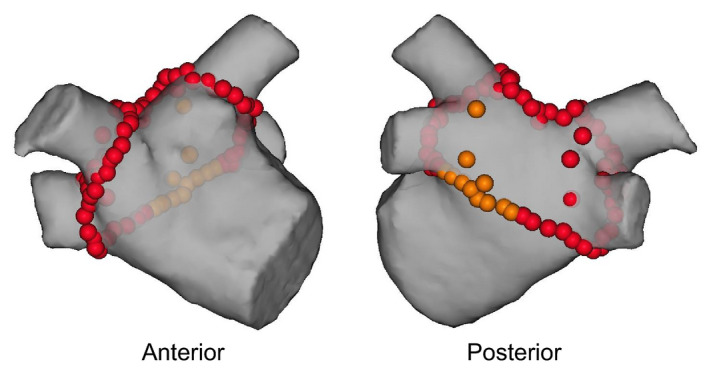
Box isolation. The red dots indicate the lesions delivered with 50 W and a target lesion size index of 5.0 (φ5 mm). The orange dots indicate the lesions on the posterior wall near the esophagus created with a delivery of 50 W for 5 s and CF of < 10 g

Ablation was started at the anterior antral wall of the left PVs in a point‐by‐point fashion. When PVs were not isolated by only anterior line, segmental ablation at the breakthrough points of the posterior portions of PVs was performed. Then, a roof line ablation was performed. Continuously, the anterior antral wall of the right PVs was ablated. Finally, a floor line ablation was performed (Figure 1). The PVs and posterior wall were mapped using a circumferential mapping catheter (Optima^TM^). When residual electrograms or gaps were detected within the box lesion using the ablation catheter or a circumferential mapping catheter, applications were added until all electrograms were eliminated.

When AF was still sustained after the BOXI, the AF was internally cardioverted. The BOXI was confirmed by high output (10 V) pacing along the ablation lines using the ablation catheter during sinus rhythm (loss of pacing capture).^3^ Additional ablation was performed at the pacing capture sites. The exit block was also confirmed by pacing within the box lesion.

Finally, the AF inducibility was evaluated by rapid pacing from the coronary sinus for 20 beats, while shortening the cycle length by 10 ms from 250 to 180 ms after isoproterenol (10 µg) injection. When non‐PV foci, atrial flutter, and atrial tachycardia were induced, they were ablated. Other extensive ablation of complex fractionated atrial electrogram or low‐voltage areas was not performed.

### 2.2 BOXI with conventional technique

All ablation applications except for those near the esophagus were delivered with 30‐40 W for 30 seconds at each site using a non‐CF sensing catheter (FlexAbility^TM^). We used a catheter irrigation rate of 8‐15 mL/min and temperature cutoff of 42°C. Ablation applications near the esophagus were delivered with 20 W and the application was stopped when the esophageal temperature increased to 40°C. The BOXI was performed in the same manner.

### 2.3 Periprocedural care and follow‐up

Anticoagulation therapy was continued before and after the ablation for 3 months in all patients. We performed transesophageal echocardiography in patients with sustained AF 1 day before the procedure. All antiarrhythmic agents were interrupted at least five half‐lives before the procedure. Amiodarone was not used in any patients. In patients with persistent and longstanding persistent AF, those antiarrhythmic drugs were re‐administered after the procedure and discontinued by 3 months (blanking period). An oral proton pump inhibitor was continued after the ablation for 1 month in all patients.

We asked a family doctor to follow‐up with the patients every month and record an electrocardiogram (ECG) every month and 24‐hour Holter monitoring every 3 months. We sent a questionnaire about AF recurrence and complications to a family doctor by fax every 3 months. We asked the patients to visit our hospital at 3, 6, and 12 months and then every 6 months. A 7‐day Holter monitoring was performed at 6 and 12 months. A telemetry ECG recorder (HCG‐801; Omron) was also used to document symptomatic episodes and to record the ECG once per week regardless of the symptoms. A recurrence was defined as any atrial arrhythmias documented by an ECG, Holter monitoring, or event recorder of >30 seconds in duration 3 months after the ablation.

### 2.4 Statistical analysis

Statistical analysis was performed using the JMP Pro software (SAS Institute Inc.). Data are given as mean ± SD and as number and percent. Continuous variables were compared using an unpaired *t* test or Wilcoxon rank‐sum test between the two groups. Categorical variables were compared between groups by a Chi‐square analysis or Fisher's exact test. Atrial tachyarrhythmia‐free survival curve was estimated using the Kaplan‐Meier method, and a log‐rank test was used for comparisons between the two groups. Tests were two‐sided and *P* < .05 was considered significant.

## 3 RESULTS

The baseline patient characteristics are listed in Table 1. There were no significant differences between the two groups. In both groups, BOXI was completely achieved in all patients.

### 3.1 Procedural data

In the HPSD group, the mean CF was 10.1 ± 2.8 g at all lesions, except for near the esophagus, and 7.6 ± 2.7 g at the lesions near the esophagus. The mean force‐time integral was 101.1 ± 16.5 gs at all lesions, except for near the esophagus, and 40.6 ± 9.6 gs at lesions near the esophagus. The mean LSI for all lesions except for near the esophagus was 5.0 ± 0.2. The mean RF time to reach a target LSI of 5.0 was 12.9 ± 2.2 seconds. There were no data on the LSI at lesions near the esophagus (50 W for 5 seconds), because it took 6 seconds to indicate the LSI.

Table 2 shows the procedure characteristics. The total RF energy in the HPSD group was significantly lower than that in the conventional group. The mean RF time for each lesion, and BOXI creation, procedure, and fluoroscopic times were significantly shorter in the HPSD group than the conventional group. The number of 10 V pacing capture sites did not differ between the two groups. The number of additional applications for residual electrograms within the box lesion was significantly smaller in the HPSD group than the conventional group. The inducibility of atrial tachyarrhythmias and the incidence of an SVC isolation and CTI ablation did not differ between the two groups. The number of RF point with esophageal temperature rise to 40°C was significantly smaller and the maximum esophageal temperature was significantly lower in the HPSD group than the conventional group.

**TABLE 2:** Procedure characteristics

	HPSD (n = 80)	Conventional (n = 80)	*P*
Total RF energy, kJ	43.0 ± 18.2	78.1 ± 24.8	<.0001
RF time per lesion, s	11.9 ± 2.4	26.5 ± 1.6	<.0001
BOX creation time, min	25.7 ± 8.3	43.6 ± 14.7	<.0001
Procedure time, min	64.7 ± 12.0	85.4 ± 19.2	<.0001
Fluoroscopic time, min	18.0 ± 4.7	22.2 ± 7.8	.0002
Additional ablation in box, n	3.9 ± 2.8	6.2 ± 3.9	<.0001
10 V pace capture site, n	10.6 ± 5.5	9.4 ± 6.2	.223
Inducibility, n (%)	17 (21.3)	20 (25.0)	.708
SVC isolation, n (%)	12 (15.0)	18 (22.5)	.311
CTI ablation, n (%)	14 (17.5)	16 (20.0)	.840
RF point with ET> 40°C, n	1.0 ± 0.5	2.7 ± 1.7	<.0001
Max ET, °C	40.4 ± 1.2	41.2 ± 0.9	<.0001

Abbreviations: CTI, cavotricuspid isthmus; ET, esophageal temperature; HPSD, high‐power short‐duration; RF, radiofrequency; SVC, superior vena cava.

### 3.2 Complications

There were no instances of esophageal injury (gastrointestinal symptoms, gastric hypomotility, or atrioesophageal fistula), pericardial tamponade, strokes or transient ischemic attacks, PV stenosis, or death in either group.

### 3.3 Clinical outcomes

In the HPSD group, 11 (13.8%) patients had a recurrence of an atrial tachyarrhythmia, including AF in eight patients, atrial flutter in 1, and both in 2, during a mean follow‐up of 14.3 ± 3.0 months after a single procedure. In the conventional group, 19 (23.8%) patients had a recurrence of an atrial tachyarrhythmia, including AF in 14 patients, atrial flutter in 3, and both in 2, during a mean follow‐up of 19.1 ± 2.0 months after a single procedure.

**FIGURE 2:**
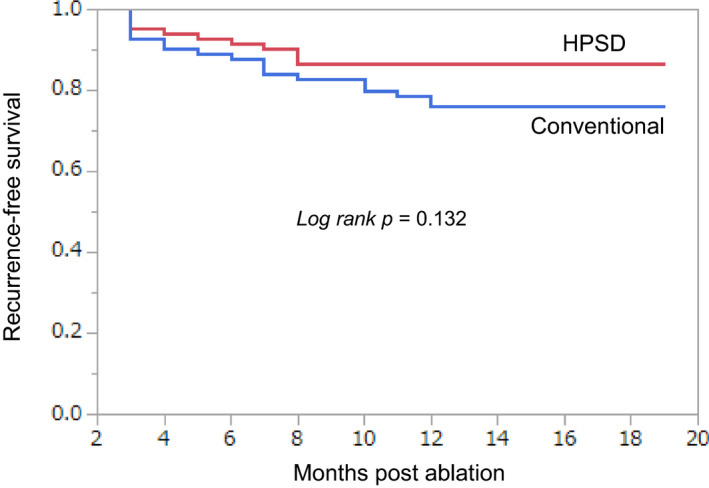
Kaplan‐Meier survival curves of the freedom from an atrial tachyarrhythmia recurrence after a single ablation procedure for the comparison between the ablation strategies (high‐power short‐duration: high‐power, short‐duration [HPSD] vs conventional technique)

Figure 2 shows the Kaplan‐Meier survival curves for the freedom from an atrial tachyarrhythmia recurrence for the two groups. The atrial tachyarrhythmia‐free rate at 12‐months after a single procedure was 86.3% (86.7% for paroxysmal, 87.0% for persistent, and 85.2% for longstanding AF) in the HPSD group and 76.3% (83.3% for paroxysmal, 76.0% for persistent, and 71.0% for longstanding AF) in the conventional group, respectively (*P* = .132).

### 3.4 PV reconnections and line gaps during the second procedure

A second procedure was performed in seven patients in the HPSD group and 14 in the conventional group (Table 3). In the HPSD group, four of seven patients had the PV reconnections in nine of 28 PVs, five patients had roof line gaps, and five had floor line gaps. In the conventional group, 10 of 14 patients had PV reconnections in 21 of 56 PVs, 11 patients had roof line gaps, and 9 had floor line gaps (Table 3). The incidence and distribution of PV reconnections and gaps in the roof or floor lines did not differ between the two groups (Table 3).

**TABLE 3:** PV reconnection and line gaps during the second procedure

	HPSD (n = 80)	Conventional (n = 80)	*P*
Second procedure n (%)	7 (8.8)	14 (17.5)	.159
PV reconnection, n (%)	4 (57.1)	10 (71.4)	.160
Total, n (%)	9 (32.1)	21 (37.5)	.810
LSPV, %	22.2	28.6	1.000
LIPV, %	11.1	28.6	.354
RSPV, %	44.4	23.8	.336
RIPV, %	22.2	19.0	1.000
Roof line gap, n (%)	5 (71.4)	11 (78.6)	1.000
LSPV side, %	35.7	37.5	1.000
Mid, %	28.6	33.3	1.000
RSPV side, %	35.7	29.2	.642
Floor line gap, n (%)	5 (71.4)	9 (64.3)	1.000
LIPV side, %	28.6	38.1	1.000
Mid, %	35.7	33.3	.642
RIPV side, %	35.7	28.6	.362

Abbreviations: AF, atrial fibrillation; HPSD, high‐power short‐duration; PV, pulmonary vein; LSPV, left superior PV; LIPV, left inferior PV; RSPV, right superior PV; RIPV, right inferior PV.

## 4 DISCUSSION

### 4.1 Main findings

The present study demonstrated that (a) BOXI with HPSD ablation is effective, feasible, and safe with short BOXI creation, procedure, and fluoroscopic times and small amount of the total RF energy deliveries; and (b) the number of pacing capture sites, atrial tachyarrhythmia‐free rate, and incidence of PV reconnections and line gaps were similar between the HPSD and conventional groups.

### 4.2 Beneficial effects of the BOXI in persistent AF

Previous studies have shown that BOXI in addition to the PVI results in a better outcome than a PVI alone or PVI plus a roof line ablation in patients with persistent AF.^4^, ^5^, ^6^, ^7^, ^8^, ^9^, ^10^, ^11^, ^12^ The BOXI can capture the triggers, rotors, and low‐voltage areas in the posterior LA,^5^ facilitate AF termination and its noninducibility,^5^ and decrease the rotors and multiple wavelets on the anterior wall, inferior wall, and LA appendage.^20^

### 4.3 Safety of the HPSD ablation on the posterior wall

An animal study has suggested that HPSD (50 W for 5 seconds) ablation has lower complication rates than LPLD ablation (40 W for 30 seconds).^21^ Winkle et al^16^ demonstrated that the incidence of an atrioesophageal fistula is significantly less common during HPSD (45‐50 W for 2‐10 seconds) ablation than LPLD (35 W for 20 seconds) ablation (0.009% vs 0.12%, *P* = .021). Baher et al^22^ evaluated esophageal injury using late gadolinium enhancement magnetic resonance imaging and demonstrated that the incidence and severity of the esophageal injury were similar between the HPSD (50 W for 5 seconds) and LPLD (≤35 W for 10‐30 seconds) procedures. In contrast, Bunch et al^18^ reported that HPSD ablation (50 W for 2 seconds) increased the recurrence of atrial flutter as compared to LPLD ablation. Thus, 50 W for 2 seconds may be too short to create effective transmural lesions and 50 W for 5 seconds may be safe for the posterior wall. Therefore, we used a setting of 50 W for 5 seconds on the posterior wall as a requisite minimum for forming adequate lesions, while minimizing the risk of esophageal injury. In the present study, the number of RF point with esophageal temperature rise to 40°C was significantly smaller and the maximum esophageal temperature was significantly lower in the HPSD group than the conventional group. Thus, the risk of esophageal injury may be minimized even with HPSD ablation as far as a short duration of 5 seconds and a reduced CF of <10 g is used near the esophagus.

### 4.4 Advantages of the LSI‐guided HPSD ablation

The LSI is multiparametric index that incorporates RF time, CF, and power, therefore, a high‐power application can reach the target LSI in a shorter time. We also showed that a 50 W RF application reached a target lesion (LSI of 5.0) within a mean of 12.9 seconds at each site. Therefore, the HPSD ablation shortened the BOXI creation, procedure, and fluoroscopic times without reducing the acute and chronic effects in terms of the number of pacing capture sites, atrial tachyarrhythmia‐free rate, and incidence of PV reconnections and line gaps. That is meaningful because longer ablation times increase the incidence of heart failure due to an increased fluid load^23^ and cognitive dysfunction.^24^ Also, shorter procedure and fluoroscopic times are preferable for patients, operators, and the staff.

### 4.5 Study limitations

First, this was a single‐center study and the number of patients was too small to evaluate for the safety issue. Second, we did not perform esophagogastroscopies to assess esophageal injury after ablation, because no patients had significant gastrointestinal symptoms. Therefore, we cannot deny the gastric hypofunction and asymptomatic esophageal lesions. Third, it was possible that asymptomatic recurrences were undetected. Finally, the follow‐up period was relatively short. Further long‐term follow‐up studies are necessary.

## 5 CONCLUSIONS

Box isolation with HPSD ablation is effective, feasible, and safe and provides a shorter procedure time and smaller amount of total RF energy deliveries without reducing the clinical outcomes.

## CONFLICT OF INTEREST

Authors declare no conflict of interests for this article.
